# Trauma and post-traumatic stress disorder in children and adolescents

**DOI:** 10.1080/20008198.2017.1351198

**Published:** 2017-09-29

**Authors:** Gerasimos Kolaitis

**Affiliations:** ^a^ Department of Child Psychiatry, Medical School, National and Kapodistrian University of Athens, ‘Aghia Sophia’ Children’s Hospital, Athens, Greece

**Keywords:** Trauma, post-traumatic stress disorder (PTSD), children and adolescents

## Abstract

Many children and adolescents are exposed to different types of trauma, e.g. abuse or various disasters. Trauma can cause severe and long-term impairment and consequences, the most studied of which are post-traumatic stress disorder (PTSD) and PTSD symptoms (PTSS). PTSD is highly prevalent in clinical practice (about 7%) and is a debilitating consequence of trauma. Of those children and adolescents exposed to trauma, about 16% will develop PTSD: almost 10% as a consequence of non-interpersonal traumatic events and 25% following interpersonal traumas. In this paper, we review predictors, assessment and treatment options for youth with PTSD (symptoms) and give directions for future research.

In the Diagnostic and Statistical Manual of Mental Disorders, 5th Edition (DSM-5), PTSD has been included in the new chapter on Trauma- and Stressor-Related Disorders and now also includes a subtype of PTSD for preschool children; this represents a significant step in DSM taxonomy as it is the first developmental subtype of a psychiatric disorder. More emphasis has been placed on behavioural changes, with new wording, and consequently the chances of diagnosing PTSD in this population have been enhanced three- to eight-fold.

Predictors of PTSD include acute stress reaction, depression, anxiety, parental effects, and smaller effects of female gender, injury severity, duration of hospitalization, heart rate after admission, pre-existing psychiatric problems, history of significant losses or threat to life, insufficient psychological and social support systems, and presence of functional impairment. Other consequences of trauma include depression, anxiety, addiction and somatic health problems.

The thorough and accurate assessment of trauma and its impact using the appropriate instruments is important to implement appropriate early prevention and treatment interventions (Olff, 2015). The study of phenotypes or domains, e.g. cognitive, memory and executive functioning, may be a new approach in studying PTSD and its impact.

There are few studies on the long-term effects of mass trauma on victimized communities (Thordardottir et al., 2016). In the aftermath of major natural disasters, acute stress reactions are expected, and overall resilience is the rule rather than the exception. Many studies have shown that 1–6 months post-trauma, PTSD is reduced by approximately 50%; nevertheless, there are doubts as to whether there is further reduction of PTSD after 6 months post-trauma.

A large recent meta-analysis showed that psychotherapy for PTSD symptoms has a small or large effect size depending on the control group; cognitive behavioural therapy has the highest effect sizes, especially in individual therapy with parental involvement (Gutermann et al., 2016). Key components of effective treatment are psychoeducation about trauma reactions, exposure to trauma-related cues and memories until they become habituated, coping skills training for children to help them to manage their anxiety, and parental training to help them to facilitate their children’s recovery. Medications such as selective serotonin reuptake inhibitors are used to treat disturbing PTSD symptoms and comorbidity, and to facilitate psychotherapy. However, more research is needed into their efficacy and safety in this vulnerable population.

In summary, we are still at the beginning of research on trauma and PTSD in children and adolescents. We need more studies of better quality, longitudinal studies and modified psychotherapies to meet younger patients’ needs. The same is true for a possible role of early pharmacotherapy (e.g. opiates, beta-adrenergic blockers) in reducing or preventing PTSD symptoms. Evaluation of both biological and psychosocial predictors that increase the risk of later development and maintenance of PTSD is important for early prevention and treatment. It has been suggested that we should use a dimensional rather than a categorical clinical entity of PTSD, and/or approach trauma beyond PTSD but also in terms of resilience and post-traumatic growth, beyond single predictors and linear associations, beyond the individual level (family is considered more and more important) and also in terms of a developmentally oriented theory. Without treatment, PTSD can become chronic and have an impact on normal psychosocial development and functioning in adulthood. Therefore, there is a need for action and a public health approach with regard to children’s traumatic exposure. The role of national and international organizations (e.g. the International and European Societies for Traumatic Stress Studies) could be important.
